# Usefulness of Intra-Aortic Balloon Pump Off Test With Echocardiography for Decision Making in Secondary Ischemic Mitral Regurgitation: A Case Report

**DOI:** 10.3389/fcvm.2022.790098

**Published:** 2022-02-07

**Authors:** Noriko Shiokawa, Masaki Izumo, Shingo Kuwata, Yoshihiro J. Akashi

**Affiliations:** ^1^Department of Ultrasound Center, St. Marianna University Hospital, Kawasaki, Japan; ^2^Division of Cardiology, Department of Internal Medicine, St. Marianna University School of Medicine, Kawasaki, Japan

**Keywords:** mitral regurgitation, heart failure, intra-aortic balloon pump, transcatheter mitral valve repair, echocardiogaphy

## Abstract

Our patient was a 60-year-old male with myocardial infarction. Urgent percutaneous coronary intervention was performed with intra-aortic balloon pump (IABP) support. Despite successful revascularization, the patient suffered from cardiogenic shock and heart failure. Secondary mitral regurgitation (MR) was mild and seemed unlikely to be the cause of heart failure. However, when IABP was temporarily stopped (IABP-OFF), secondary MR was aggravated; therefore, we decided to perform transcatheter mitral valve repair. Thereafter, only mild residual MR was observed after IABP removal, and hemodynamic stability was achieved. This case presents IABP-OFF test with echocardiography as a useful method to assess secondary MR.

## Introduction

**Figure 1:**
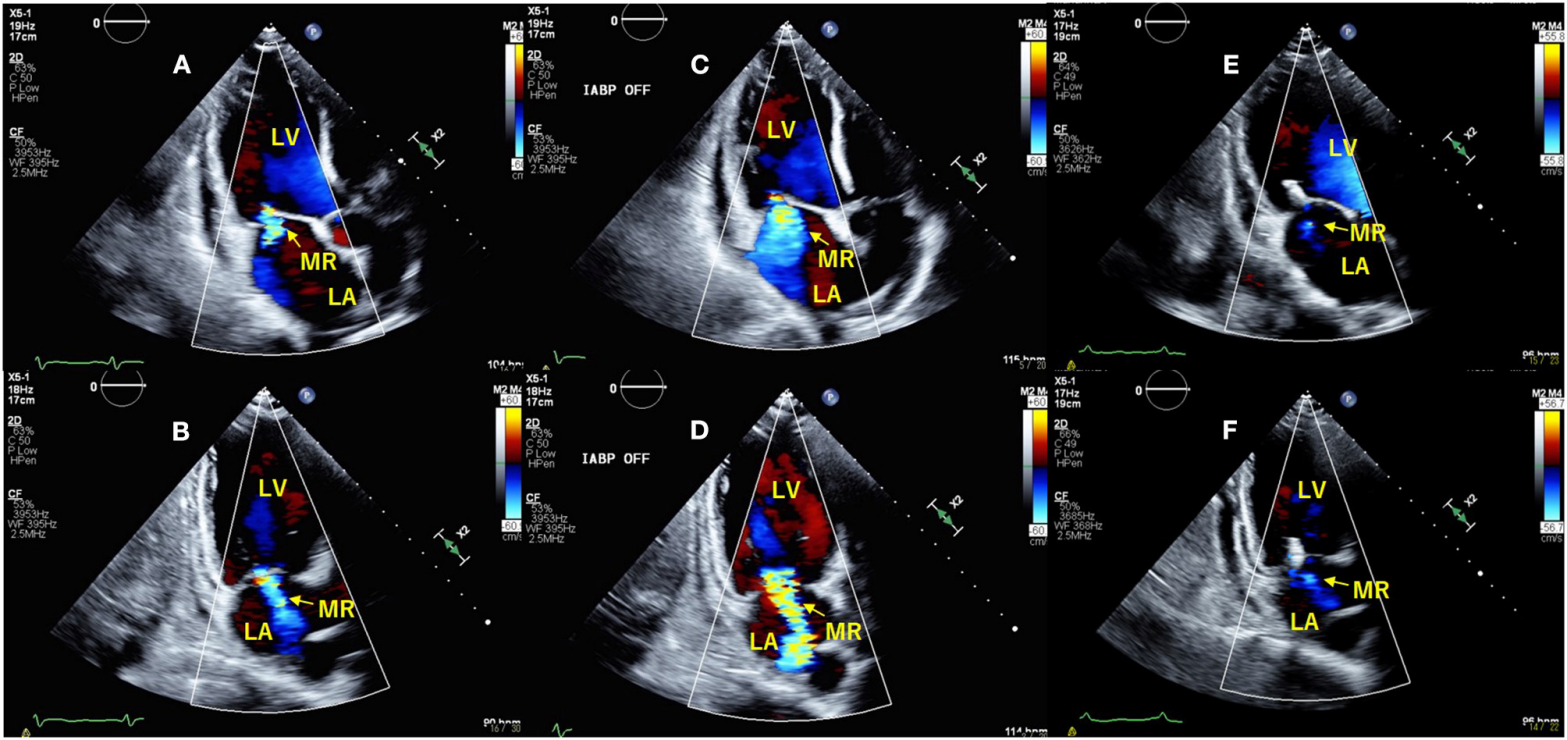
Transthoracic echocardiography with intra-aortic balloon pump (IABP)-ON **(A,B)** and -OFF **(C,D)**. The apical long-axis view **(A,C)** and two-chamber view **(B,D)** show that mitral regurgitation is significantly increased from IABP-ON to IABP-OFF. After intervention with the MitraClip™, secondary MR was significantly decreased **(E,F)**. LV, left ventricle; LA, left atrium.

A 60-year-old male with a history of hypertension and dyslipidemia was transported to another hospital due to chest pain and loss of consciousness. On admission, electrocardiogram showed ST elevation in leads V 1 ~ 5, II, III, and aVF. Emergency coronary angiography was performed under the diagnosis of ST elevation myocardial infarction. Coronary angiography showed complete occlusion of the left main trunk, it was diagnosed as a culprit lesion and primary PCI was performed under IABP support. Drug eluting stent was placed from the left main trunk to the left anterior descending artery. Upon urgent coronary angiography, acute myocardial infarction of the main trunk of the left coronary artery was diagnosed. Approximately 1 month after the primary PCI, heart failure and cardiogenic shock were observed at the previous hospital. Percutaneous intervention (PCI) was then urgently performed with intra-aortic balloon pumping (IABP) support. The patient was transferred to our hospital with IABP inserted to treat cardiogenic shock and heart failure. We performed IABP on/off test with echocardiography the day after he was transferred to our hospital. Transthoracic echocardiography (TTE) revealed the following: left ventricular ejection fraction (LVEF), 32%; left ventricular end-diastolic volume (LVEDV), 175 mL; left ventricular end-systolic volume (LVESV), 120 mL; and left atrial volume index (LAVi), 55 mL/m^2^. The severity of secondary mitral regurgitation (MR) was mild to moderate (Figures 1A,B, Supplementary Videos 1, 2) under IABP support, with an effective regurgitant orifice area (EROA) of 0.24 cm^2^, regurgitant volume (RV) of 16 mL, and vena contracta width (VCW) in two-chamber view of 4.6 mm. The coaptation length of the mitral valve was 2.9 mm, and the mitral annular diameter was 29.6 mm. IABP support was temporarily stopped (IABP-OFF) to assess possible changes in the patient's status. The coaptation length shortened to 1.7 mm, and the mitral annular diameter increased to 32.5 mm. MR significantly worsened with EROA of 0.37 cm^2^, RV of 29 ml, and VCW of 12.7 mm; this indicated MR aggravation compared to what was observed on IABP-ON (Figures 1C,D, Supplementary Videos 3, 4). The velocity time integral at the left ventricular outflow tract was 8 cm in IABP-ON and 7 cm in IABP-OFF; thus, a decrease in forward cardiac output was evident. Based on these findings, we concluded the need for intervention to secondary MR. We decided to perform mitral valve intervention by a transcatheter edge-to-edge repair with a MitraClip™ (Abbott Vascular,. Abbott Park, IL). The G4-XTW was placed in the A2P2 region, resulting in a reduction of MR to the degree of trivial MR. The hemodynamics, including the left atrial pressure, also improved to be within the tolerance of the mitral valve mean pressure gradient of 3 mm Hg. After transcatheter mitral valve repair with the MitraClip™, only mild residual MR (Figures 1E,F, Supplementary Videos 5, 6) was observed after removal of the IABP, and hemodynamic stability was achieved. Intraprocedural transesophageal echocardiography showed mild secondary MR even after discontinuation of IABP. We inserted a pigtail catheter into the left atrium during MitraClip, and performed the procedure while monitoring the LA pressure (1). The mean LA pressure decreased from 44 to 33 mmHg before and after MitraClip. IABP was removed during the operation. After the MitraClip, heart failure and cardiogenic shock improved, and the patient was discharged 8 days after the MitraClip.

## Discussion

This is a case of acute myocardial infarction complicated by cardiogenic shock, wherein the IABP-OFF test revealed that secondary MR was one of the causes of cardiogenic shock and heart failure. The closure position of the mitral valve leaflet is normally determined by the balance between the closing force from the left ventricular pressure during systole and the tethering force from the chordae tendineae. However, in secondary MR, remodeling of the left ventricle occurs after myocardial infarction, and the valve leaflet shifts posteriorly and/or apically due to myocardial damage around the papillary muscle. This causes valvular apex malfunction, enhanced tethering, and exacerbated MR in patients with dilated left ventricle and decreased left ventricular function (2). Compared with chronic secondary MR, loss of mitral valve coaptation and hemodynamic loading in the acute phase may result in significant secondary MR despite relatively small mitral valve tethering (3). In IABP support, the balloon inflates during diastole to increase diastolic pressure and coronary flow. During systole, the balloon contracts, reducing ventricular afterload, and myocardial oxygen consumption. This effect results in temporary reverse remodeling of the left ventricle, improved mitral coaptation, and decreased MR. Eliaz et al. reported that reduced mitral annulus diameter and increased mitral valve leaflet coaptation were observed in patients with severe MR and with IABP support (4). In this case, IABP-OFF resulted in an expanded mitral annulus diameter, shortened coaptation length, and increased MR. As far as we know, this case is the first case of utility of IABP off-test for decision making of secondary MR. Further studies are needed to investigate the severity assessment of secondary MR using IABP off-test with echocardiography.

## Conclusions

This case shows that the IABP ON-OFF test with transthoracic echocardiography is a non-invasive method that can evaluate the severity of MR and the morphology of the mitral valve in patients with difficulty in weaning from IABP support. It is considered to be a useful method for diagnosing the severity of secondary MR and deciding whether to perform mitral valve intervention.

## Data Availability Statement

The original contributions presented in the study are included in the article/Supplementary Material, further inquiries can be directed to the corresponding author/s.

## Author Contributions

NS and MI contributed data collection and writing the manuscript. SK and YA contributed to the critical revision of the manuscript. All authors contributed to the article and approved the submitted version.

## Conflict of Interest

MI and SK are training faculty of Abbott Medical Japan. The remaining authors declare that the research was conducted in the absence of any commercial or financial relationships that could be construed as a potential conflict of interest.

## Publisher's Note

All claims expressed in this article are solely those of the authors and do not necessarily represent those of their affiliated organizations, or those of the publisher, the editors and the reviewers. Any product that may be evaluated in this article, or claim that may be made by its manufacturer, is not guaranteed or endorsed by the publisher.

## Supplementary Material

Supplementary Video 1 The apical long axis view with intra-aortic balloon pump (IABP)-ON.

Supplementary Video 2 The two-chamber view with IABP-ON.

Supplementary Video 3 The apical long axis view with IABP-OFF.

Supplementary Video 4 The two-chamber view with IABP-OFF.

Supplementary Video 5 The apical long axis view after transcatheter mitral valve repair.

Supplementary Video 6 The two-chamber view after transcatheter mitral valve repair.
